# Efficacy and safety of acupuncture combined with analgesics on lung cancer pain: A protocol for systematic review and meta-analysis

**DOI:** 10.1097/MD.0000000000026225

**Published:** 2021-06-11

**Authors:** Renqing Kuang, Guojiang Xiong, Wei Lv, Yun Zhao, Min Yu, Jiawang Jiang

**Affiliations:** a Jiangxi University of Chinese Medicine; b The Affiliated Hospital of Jiangxi University of Chinese Medicine, Nanchang, China.

**Keywords:** acupuncture, lung cancer, pain, protocol, systematic review

## Abstract

**Background:**

Lung cancer (LC) is the malignant tumor with the highest incidence in the world, and treatment methods include surgery, radiotherapy, chemotherapy, and immunotherapy. Cancer pain is a common symptom in patients with LC, and the clinical treatment is to relieve it with analgesics. Acupuncture can relieve cancer pain. This study aims to systematically study the efficacy and safety of acupuncture combined with analgesics on cancer pain in patients with LC.

**Methods:**

From the beginning to April 2021, search Medline, Embase, Cochrane Central Controlled Trials Register (Central), China National Knowledge Infrastructure (CNKI), Wanfang Database, China Biomedical Literature Database (CBM), and Chinese Science Journal Database (VIP database). Search the international clinical trial registration platform and the Chinese clinical trial registration platform to find ongoing or unpublished trials. The main outcome indicator is the total effective rate of analgesia, and the secondary outcome indicator is pain intensity score and adverse reactions. The RevMan 5.4 software will be used for statistical analysis.

**Results:**

This study will provide the latest evidence for acupuncture combined with analgesics to relieve LC pain.

**Conclusion:**

The conclusion of this study is to evaluate the effectiveness and safety of acupuncture combined with analgesics in alleviating LC pain.

**INPLASY registration number:**

INPLASY202150051

## 1 Introduction

Primary bronchial lung cancer, also known as lung cancer (LC), ranks second in absolute incidence globally and in developing countries, and is the most common cause of cancer death in the world.^[1]^ Living environment and social lifestyle are related to the occurrence of LC. Smoking is almost one of the important factors in the development of LC.^[2]^ The current main treatments for LC include surgery, chemotherapy, radiotherapy, and targeted therapy, etc.^[3]^ Patients with LC reported a series of physical and psychological social symptoms, including coughing, dyspnea, pain, and anxiety.^[4]^ Pain is one of the most serious symptoms of LC patients^[5]^ and seriously affect the patient's quality of life.^[6]^ The cause of LC pain is related to the location of the tumor, the spread and metastasis of the tumor, and the treatment plan.^[7,8]^ Regarding LC pain, the current main treatment is the use of analgesics, but studies have shown that opioids can cause hypogonadism, inhibit the hypothalamic-pituitary-adrenal axis, and have an impact on bone health.^[9]^ Patients with significant pain are more likely to use alternative therapies such as massage, acupuncture, herbal medicine, and hyperthermia.^[10]^

As a traditional treatment method, acupuncture has been used clinically, and it may be a better choice for the treatment of LC pain.^[11]^ A large number of studies have shown that acupuncture can be effective for patients with pain symptoms in cancer patients.^[12]^ The main mechanism of action is to activate the pain modulation system in the body, regulate the human nerve-endocrine-immune system, activate the production, and release of endogenous opioids,^[13]^ and at the same time promote the reduction of substance P and the increase of β-EP content, to achieve the purpose of alleviating cancer pain.^[14]^ In addition, related studies have shown that acupuncture can reduce postoperative opioid consumption and improve postoperative analgesia.^[15]^

In recent years, some research data have shown that acupuncture can relieve cancer pain. Although some existing studies have shown that acupuncture combined with analgesics can improve LC pain, there is a lack of relevant comprehensive evaluation. Therefore, we decided to conduct this study to further evaluate the efficacy and safety of acupuncture combined with analgesics in patients with LC pain and provide clinical evidence.

## 2 Objectives

The aim of this study was to evaluate the efficacy and safety of acupuncture combined with analgesics on LC pain, and provide the latest evidence of evidence-based medicine for the clinical treatment of LC pain.

## 3 Methods and analysis

### 3.1 Study protocol and registration

The review scheme of this study has been registered on the INPLASY website (registration number: INPLASY202150051 https://inplasy.com/inplasy-2021-5-0051/). This protocol will be reported in accordance with the Preferred Reporting Project (PRISMA-P) Statement Guidelines for Systematic Reviews and Meta-Analysis Agreements.^[16]^

### 3.2 Inclusion criteria

#### 3.2.1 Types of studies

All randomized clinical trials (RCTs) of acupuncture combined with analgesics in the treatment of LC pain, whether blinded or unblinded.

#### 3.2.2 Type of participants

All the patients included in the study were diagnosed LC pain, regardless of age, sex, race, and course of disease.

#### 3.2.3 Type of interventions

The treatment plan is acupuncture combined with analgesics. Acupuncture treatment includes all types, such as electric acupuncture, fire acupuncture, warm acupuncture, body acupuncture, and so on.

#### 3.2.4 Type of comparators

Conventional analgesic treatment.

#### 3.2.5 Types of outcome measures

The main outcome measure was the total effective rate of analgesia (excluding the evaluation of inefficiency and non-remission rate). The secondary outcome indicators were pain intensity score and adverse reactions.

### 3.3 Exclusion criteria

1. RCTs comparing 2 different types of needle knife;
2. Non-randomized controlled trials;
3. Duplicated data;
4. Invalid outcome indexes.

### 3.4 Study search

The English databases include PubMed, Embase, Web of Science, Cochrane Library, and Chinese databases include China National Knowledge Infrastructure (CNKI), Wanfang Data, VIP Database (VIP), China Biomedical Literature (CBM). From the establishment of the database to April, 2021, the key words include “acupuncture,” “body acupuncture,” “electro-acupuncture,” “warm acupuncture,” “auricular acupuncture,” “fire needling,” “analgesics,” “lung cancer,” “pain.” In addition, we will also retrieve ongoing or unpublished trials from the International Clinical Trial Registration Platform and Chinese Clinical Trial Registry Platform. PubMed's search strategy is shown in Table 1.

**Table 1:** Search strategy used in PubMed database.

Order	Search items
#1	(((((((((((((((((((((((((((Pain) OR (Pain, Burning)) OR (Burning Pain)) OR (Burning Pains)) OR (Pains, Burning)) OR (Suffering, Physical)) OR (Physical Suffering)) OR (Physical Sufferings)) OR (Sufferings, Physical)) OR (Pain, Migratory)) OR (Migratory Pain)) OR (Migratory Pains)) OR (Pains, Migratory)) OR (Pain, Radiating)) OR (Pains, Radiating)) OR (Radiating Pain)) OR (Radiating Pains)) OR (Pain, Splitting)) OR (Pains, Splitting)) OR (Splitting Pain)) OR (Splitting Pains)) OR (Ache)) OR (Aches)) OR (Pain, Crushing)) OR (Crushing Pain)) OR (Crushing Pains)) OR (Pains, Crushing)) AND ((((((((((((((((((Lung Neoplasms) OR (Pulmonary Neoplasms)) OR (Neoplasms, Lung)) OR (Lung Neoplasm)) OR (Neoplasm, Lung)) OR (Neoplasms, Pulmonary)) OR (Neoplasm, Pulmonary)) OR (Pulmonary Neoplasm)) OR (Lung Cancer)) OR (Cancer, Lung)) OR (Cancers, Lung)) OR (Lung Cancers)) OR (Pulmonary Cancer)) OR (Cancer, Pulmonary)) OR (Cancers, Pulmonary)) OR (Pulmonary Cancers)) OR (Cancer of the Lung)) OR (Cancer of Lung))[All Fields]
#2	((((((((((((((((Acupuncture) OR (acupuncture therapy)) OR (Electroacupuncture)) OR (electroacupuncture therapy)) OR (manual acupuncture)) OR (moxibustion)) OR (Acupuncture, Ear)) OR (Acupunctures, Ear)) OR (Ear Acupunctures)) OR (Auricular Acupuncture)) OR (Ear Acupuncture)) OR (Acupuncture, Auricular)) OR (Acupunctures, Auricular)) OR (Auricular Acupunctures)) OR (warm acupuncture)) OR (fire needling)) OR (elongated needle)[All Fields]
#3	((((((((Analgesics) OR (Anodynes)) OR (Analgesic Drugs)) OR (Drugs, Analgesic)) OR (Analgesic)) OR (Analgesic Agents)) OR (Agents, Analgesic)) OR (Antinociceptive Agents)) OR (Agents, Antinociceptive)[All Fields]
#4	randomized controlled trial [Publication Type] OR randomized [Title/Abstract] OR placebo [Title/Abstract]
#5	#1 AND #2 AND #3 AND #4

### 3.5 Selection of studies and data extraction

#### 3.5.1 Studies selection

**Figure 1:**
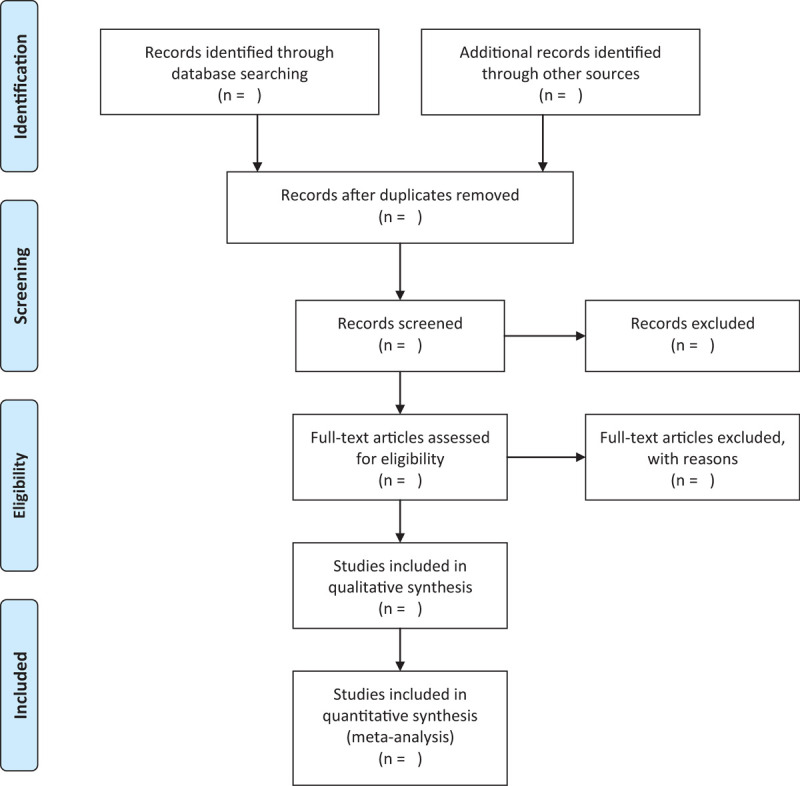
Flow chart of study selection.

The literature search results were independently completed by 2 examiners according to the inclusion criteria. Import all the documents searched in the database into EndNote X9 to exclude duplicate documents. Exclude obviously unqualified articles by reading the title and abstract, then read the full text of the remaining articles, and discuss the final included documents in groups. When there is a difference in the results of the literature screening, it will be discussed and resolved with the third examiner. The selection process will be shown in the PRISMA flow chart (Fig. 1).

#### 3.5.2 Data extraction

The 2 researchers used predesigned tables to collect data, collect the following information: study data (first author or newsletter author, publication time, study type), subject details (baseline data, sex, age), research methods (sample size, randomization, blind method, distribution concealment, and so on), intervention measures in the treatment group and the control group, acupuncture therapy, result measurement (primary and secondary results, adverse events). Any differences in the data collection process will be resolved through discussion by a third researcher. If the above data in the article is incomplete, we will contact the author to supplement it.

### 3.6 Risk of bias assessment

Two reviewers will use Cochrane collaborative tools to evaluate the quality of the literature.^[17]^ It includes the following 7 domains: random sequence generation, allocation concealment, blinding of participants and personnel, blinding of outcome assessment, incomplete outcome data, selective reporting, and other sources of bias. Each domain includes a judgment of low, high, and unclear risk of bias according to information provided by authors. Disagreements between reviewers will be resolved through discussion with a third reviewer.

### 3.7 Data synthesis and statistical methods

#### 3.7.1 Data synthesis

For continuous data, will be used mean difference (MD) as the effect indicator with 95% confidence interval, and dichotomous data will be calculated as risk ratio (RR) or odds ratio (OR) as the effect index with 95% confidence interval. If the studies are with no statistical homogeneity, the fixed-effect model can be used for analysis; if the studies are with significant statistical heterogeneity, random-effects model analysis will be used. The Review Manager (RevMan) V.5.3 software will be used for statistical analysis.

#### 3.7.2 Assessment of heterogeneity

*χ*^2^ test was used to estimates the degree of heterogeneity. When *I*^*2*^ < 40%, it indicates that no significant statistical heterogeneity. When 40% < *I*^*2*^ < 75%, it indicates that there is statistics heterogeneity, and the source of heterogeneity needs to be further analyzed. When *I*^*2*^ > 75%, it indicates that there is statistics heterogeneity between the studies.

#### 3.7.3 Subgroup analysis

Subgroup analysis will be performed if there have heterogeneity between the study results, following items will be considered: type of acupuncture, cancer stage, sex, and age.

#### 3.7.4 Sensitivity analysis

We will eliminate the “high-risk” low-quality articles for sensitivity analysis to judge the robustness of the results.

#### 3.7.5 Grading the quality of evidence

Two researchers used the Recommendation, Evaluation, Development, and Evaluation system to independently evaluate the quality of evidence for each result, which system divides the level of evidence into 4 levels: high, medium, low, and very low.^[18]^

#### 3.7.6 Publication bias

When >10 trials were included in this study, the funnel chart was used to judge the report bias. I

### 3.8 Ethics and dissemination

The study does not involve the personal information of participants, so no ethical approval is required, and the research will be published in a peer-reviewed journal or related journal meeting.

## 4 Discussion

LC is a global health burden. Due to the limited conditions for lung cancer screening,^[19]^ this disease has almost become the most widespread and the highest mortality disease in the world. Tumor invasion makes LC pain the most common symptom of patients, which seriously affects the quality of life of patients.^[5,6]^ At present, common clinical treatments for LC include surgery, chemotherapy, radiotherapy, and targeted therapy, but opioid analgesics are still used for LC pain. Opioids have a good analgesic effect, but their adverse reactions and dependence have always been a problem that has not been effectively solved, and cancer patients are easily tolerated by analgesics.^[20]^ Acupuncture as a traditional Chinese medical technique is widely used in modern clinical practice. A large number of research reports have shown that acupuncture can effectively relieve pain. Its main mechanism is to stimulate the central nervous system to regulate inflammation and significantly reduce the expression of tumor necrosis factor-α, interleukin (IL)-1β, and IL-6.^[21]^

So far, although some RCTs have shown that acupuncture therapy combined with analgesics is effective for LC pain, there is no systematic review. Therefore, this study conducted a systematic review and meta-analysis of acupuncture combined with analgesics in the treatment of LC pain, aiming to provide the latest evidence for the treatment of LC pain and guide clinical decision-making.

## Author contributions

**Conceptualization:** Renqing kuang, Guojiang xiong.

**Data curation:** Yun Zhao, Wei Lv, Jiawang Jiang.

**Formal analysis:** Guojiang Xiong, Wei Lv.

**Methodology:** Guojiang xiong, Yun Zhao.

**Software:** Wei Lv, Yun Zhao.

**Supervision:** Guojiang xiong, Wei Lv.

**Writing – original draft:** Renqing Kuang, Jiawang Jiang, Min Yu.

**Writing – review & editing:** Renqing Kuang, Jiawang Jiang.
